# Assessment of Serum Zinc Levels in Patients With Decompensated Cirrhosis of the Liver and Its Association With Disease Severity and Hepatic Encephalopathy: A Prospective Observational Study From North India

**DOI:** 10.7759/cureus.41207

**Published:** 2023-06-30

**Authors:** Vansh Deep, Shankerdeep Sondhi, Sunita Gupta

**Affiliations:** 1 General Medicine, Maharishi Markandeshwar Institute of Medical Sciences & Research, Mullana, IND

**Keywords:** hepatic encephalopathy (he), west haven scoring, serum zinc, decompensated liver cirrhosis, chronic liver disease (cld)

## Abstract

Background

Zinc, an essential trace element, plays a vital role in cellular metabolism, and the liver is the main organ responsible for its metabolism. Because serum zinc levels are found to be lowered in chronic liver diseases, it has been hypothesized to be a precipitating factor for the development of hepatic encephalopathy.

Methodology

This prospective, observational study included patients with decompensated cirrhosis of the liver who were admitted to the medical intensive care unit of a tertiary care institute in northern India between September 2021 and April 2023. The diagnosis was based on history and detailed clinical examination. The serum zinc levels of patients were estimated using atomic absorption spectrometry at admission and compared to that of healthy controls. Serum zinc levels were correlated with the severity of liver disease and hepatic encephalopathy among the cases.

Results

A total of 100 cases of decompensated cirrhosis of the liver and 50 healthy controls were included. The mean serum zinc level of the cases was 40.5 ± 10.0 µg/dL which was significantly lower than the mean serum zinc level (104.0±9.1 µg/dL) of controls (p < 0.0001). Serum zinc level was significantly lower in patients with higher grades of hepatic encephalopathy (p = 0.000). Similarly, serum zinc level was significantly reduced among patients with higher Child-Pugh and Model for End-stage Liver Disease scores.

Conclusions

Serum zinc level is significantly reduced in patients with decompensated cirrhosis of the liver, and lower serum zinc level is associated with the increased severity of the disease and higher grades of hepatic encephalopathy. In patients with decompensated cirrhosis of the liver, maintenance of adequate serum zinc levels may prevent hepatic encephalopathy.

## Introduction

India has a significant burden of liver disease as it single-handedly contributed to 18.3% of the two million global liver disease-related deaths in 2015 [1]. The contribution of chronic liver diseases (CLDs), i.e., cirrhosis and its complications, as causes of mortality in India, has been gradually increasing since 1980 compared to China, where it remains stationary and is even showing decreasing trends [2]. In contrast, the burden of CLDs is on the decline in developed countries such as the United States where there were 167,324 incident CLDs in 2017 [3].

Cirrhosis is anatomically defined as “a diffuse process with fibrosis and nodule formation” and is the result of fibrogenesis that occurs due to chronic injury to the liver [4]. Deaths from cirrhosis are continuously rising, and it was the 11th leading cause of death and 15th most common cause of morbidity, accounting for 2.2% of all deaths and 1.5% of disability‐adjusted life years globally, in 2016 [1]. In India, the common cause causes of cirrhosis are alcohol abuse and viral hepatitis [5]. Most frequent etiologies in the West include alcoholic liver disease as well as hepatitis C, although hepatitis B remains more common in the majority of Asia and Sub-Saharan Africa [6]. Detection of cirrhosis lacking an obvious etiology is unusual today due to the discovery of the hepatitis C virus in 1989 as well as non-alcoholic steatohepatitis in obese and diabetic people [6].

Hepatic encephalopathy affects 30-45% of cirrhotic patients and is a serious consequence [7]. It is a clinical condition involving reversible neurological dysfunction and can be an indication of cerebral edema, cerebral atrophy, reversible metabolic encephalopathy, or even a combination of such conditions. It is not yet clear which pathways lead to neurological impairment in hepatic disease. Such elements have a strong connection to hepatic dysfunction [8]. For hepatic encephalopathy, several triggering events have been identified, including electrolyte abnormalities, constipation, infections, and gastrointestinal bleeding. Eliminating the triggering elements as soon as possible is crucial, and most individuals can be successfully managed by only controlling the triggering event [9].

Zinc, an essential trace element in the human body, is fundamental for the normal life cycle of cells and is involved in more than 300 enzymatic systems [10]. It is a critical co-factor in the urea cycle and plays an important role in the conversion of ammonia to urea. Zinc improves the natural defense against reactive oxygen radicals, is an anti-apoptotic and anti-inflammatory agent, and is a co-factor for DNA synthesis [11,12]. In CLD, its deficiency can make patients susceptible to endotoxemia through increased intestinal permeability [13]. Hence, a deficient zinc level appears to play a role in the development of hepatic encephalopathy. Some studies have shown that zinc deficiency is commonly seen in liver cirrhosis [14-17], and reduced serum zinc level has been found to be associated with hepatic encephalopathy [17]. In cirrhosis, zinc urine excretion is frequently elevated and its absorption in the small intestine may be hindered [15]. Further, patients with CLDs, particularly those with alcoholic liver disease, frequently consume diets deficient in zinc and protein. The main zinc-binding protein, albumin, is frequently reduced in CLDs such as cirrhosis, which, in turn, can lead to hypozincemia in these conditions [15].

Because serum zinc levels are frequently decreased in liver diseases, it has been hypothesized to be a precipitating factor for the development of hepatic encephalopathy. There is a dearth of studies particularly from India which have tried to determine the correlation of serum zinc levels with hepatic encephalopathy. With this background, this study was conducted to assess serum zinc levels in patients with decompensated cirrhosis of the liver, as well as its correlation with disease severity and stages of hepatic encephalopathy in these patients.

## Materials and methods

Study design and population

This prospective, observational study was conducted between March 2021 and April 2023 at the Department of General Medicine, Maharishi Markandeshwar Institute of Medical Sciences & Research (MMIMSR), Mullana, Haryana, North India. A total of 100 patients with decompensated cirrhosis of the liver who were admitted to the medicine intensive care unit (ICU) were taken as cases, and 50 patients without any history of any liver diseases attending the general medicine outpatient department (OPD) were taken as controls. All participants underwent a detailed history and systemic examination.

Inclusion Criteria

Patients with decompensated cirrhosis of the liver who were diagnosed through a combination of clinical and biochemical examination and ultrasonography of the abdomen were included. The presence of variceal upper gastrointestinal bleeding, hepatic encephalopathy, hepatocellular carcinoma, or ascites was considered as denoting decompensated state.

Exclusion Criteria

Patients with other metabolic causes of encephalopathy, patients in altered sensorium because of a head injury or stroke, patients with psychiatric disorders, and patients in alcohol withdrawal and acute alcohol intoxication state were excluded from the study.

Study procedure

All selected patients with decompensated liver disease were enquired in detail about their present, past, family, and personal history, especially about alcohol intake, jaundice, and drug intake. All selected patients were assessed for disease severity using the Child-Pugh score [18] and Model for End-stage Liver Disease (MELD) score [19]. The Child-Pugh scoring consists of five parameters, namely, serum bilirubin, serum albumin, ascites, encephalopathy, and prothrombin time (PT)/international normalized ratio (INR). A total Child-Pugh score of 5 to 6 is considered class A (well-compensated disease), 7 to 9 is considered class B (significant functional compromise), and 10 to 15 is considered class C (decompensated disease) [18]. MELD scoring depends on three readily available laboratory variables, namely, serum creatinine, serum bilirubin, and INR. A higher score denotes a higher risk of death in the next three months. A score of 21-25 denotes low risk, 26-30 denotes moderate risk, and >30 denotes high risk of mortality [19].

Hepatic encephalopathy grading was done using the West Haven classification [20]. This grading system differentiates four grades of clinically manifest hepatic encephalopathy. In grade I, patients show a lack of attention and some subtle personality changes that are obvious to their relatives. In grade II, the most characteristic finding is the disorientation to time combined with inappropriate behavior and lethargy. In grade III, patients are stuporous but respond to stimuli. They are disoriented to place and situation and may exhibit bizarre behavior. In grade IV, patients are in a coma [20].

Serum zinc levels were estimated at the time of presentation. About 3 mL of venous blood was obtained from all patients for biochemical assessment including the zinc assay. The samples were drawn in the morning and sent to the hospital’s laboratory within an hour. The serum zinc level was estimated using atomic absorption spectrometry.

A total of 200 age-matched (±3 years) and sex-matched healthy controls in a ratio of 2:1 to the cases were recruited who had no history of any liver diseases and were attending the OPD for minor illnesses. Relevant investigations including liver function test, renal function test, complete blood count, and serum zinc levels were also performed.

Statistical analysis

Statistical analysis was performed using SPSS software version 23.0 (IBM Corp., Armonk, NY, USA). Descriptive statistics were performed. Continuous variables were summarized as mean with standard deviations, and discrete variables were summarized as frequency with percentages. The chi-square test, Student’s t-test, and analysis of variance test were applied to compare two or more groups and draw associations. Pearson’s correlation coefficient was used to determine a correlation between continuous variables. A p-value of less than 0.05 was considered statistically significant.

Ethical considerations

The study protocol received ethical approval from the Ethical Review Board of Maharishi Markandeshwar Institute of Medical Sciences & Research, Mullana (approval number: MMIMSR/NRCE/2020-2023/637). Written informed consent was taken from all participants before inclusion in the study by duly informing them about the study details and its objectives.

## Results

A total of 100 cases of decompensated chronic liver disease (DCLD)/liver cirrhosis and 200 healthy controls were prospectively recruited for the study. The mean age of the cases was 48.4 ± 9.9 years, whereas the mean age of the controls was 48.8 ± 9.8 years with no significant difference. The majority of the patients were aged 41-60 years (68%) and males (97%) (Table 1).

**Table 1:** General characteristics of patients/cases and controls.

Variables	Cases (n = 100)	Controls (n = 200)	P-value
Mean age (in years)	48.43 ± 9.91	48.81 ± 9.89	0.11
Age groups (in years), N (%)			0.91
≤30	3 (3.0)	5 (2.5)	
31–40	20 (20.0)	38 (19.0)	
41–50	34 (34.0)	69 (34.5)	
51–60	32 (32.0)	65 (32.5)	
>60	11 (11.0)	23 (11.5)	
Gender, N (%)			1.0
Male	97 (97.0)	194 (97.0)	
Female	3 (3.0)	6 (3.0)	

**Figure 1:**
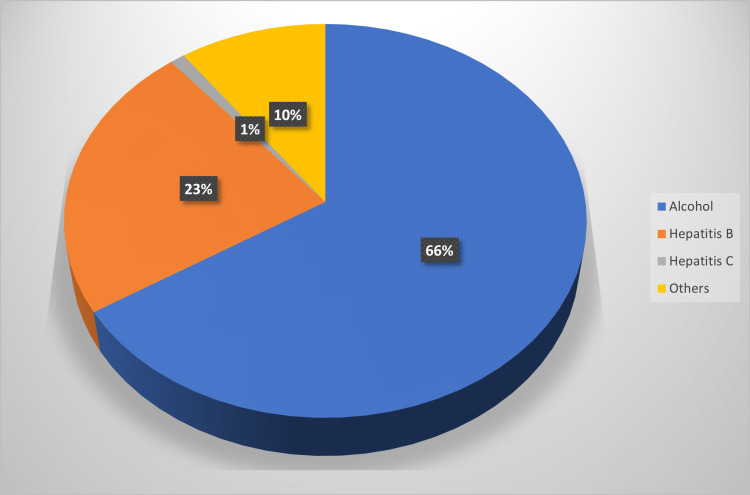
Etiological classification of patients with decompensated chronic liver disease.

With respect to the etiology of liver cirrhosis among cases, the majority were due to alcohol (66%), followed by hepatitis B infection (23%) and other causes (10%) (Figure 1).

With respect to the clinical profile of the cases, all (100%) patients had icterus/yellow discoloration of the eyes, followed by pain in the abdomen (92%), abdominal distension (92%), fever (90%), ascites (80%) and black stools (64%), with 10 (10%) patients being in a coma at the time of presentation (Table 2).

**Table 2:** Clinical profile of patients with DCLD at the time of presentation (N = 100). DCLD = decompensated chronic liver disease

Clinical profile	DCLD cases	
	Number	Percentage
Icterus/yellow discoloration of eyes	100	100.0
Abdominal distension	92	92.0
Pain in the abdomen	92	92.0
Fever	90	90.0
Ascites	80	90.0
Black stools	64	64.0
Constipation	64	64.0
Nausea/Vomiting	68	68.0
Asterixis	58	58.0
Clubbing	49	49.0
Sleep alteration	44	44.0
Hematemesis	43	43.0
Disorientation	31	31.0
Coma	10	10.0

The mean serum zinc level of the cases was 40.5 ± 10.0 µg/dL, whereas it was 104.0 ± 9.1 µg/dL for the controls. The difference was highly significant (p < 0.0001) implying that serum zinc levels of the cases were significantly lower than that of controls. A comparison of other important biochemical parameters such as serum albumin, PT/INR ratio, serum creatinine, blood urea nitrogen, and serum sodium also showed a significant difference between the two groups (Table 3).

**Table 3:** Comparison of serum zinc levels and other important biochemical parameters between cases and controls. *: Statistically significant. PT/INR = prothrombin time/international normalized ratio; BUN = blood urea nitrogen

Biochemical parameters	Cases (n = 100)	Controls (n = 200)	P-value
	Mean ± SD	Mean ± SD	
Serum zinc (µg/dL)	40.5 ± 10.0	104.0 ± 9.1	<0.0001*
Serum albumin (g/dL)	2.4 ± 0.3	4.2 ± 0.2	<0.0001*
PT/INR ratio	2.8 ± 0.2	0.9 ± 0.1	0.004*
Serum creatinine (mg/dL)	1.6 ± 0.5	1.1 ± 0.2	0.001*
BUN (mg/dL)	35.8 ± 19.3	18.2 ± 2.0	0.000*
Serum sodium (mEq/L)	134.7 ± 7.1	140.3 ± 2.4	0.04*

As per West Haven criteria for classifying hepatic encephalopathy severity, the most common was grade 2 (37%), followed by grade 1 (35%) and grade 3 (17%). On comparing the serum zinc levels of the patients across the four severity grades of HE, the difference was found to be highly significant (p = 0.000), implying that serum zinc levels significantly decreased with increasing severity of hepatic encephalopathy (Table 4).

**Table 4:** Classification of the DCLD patients per the severity grades of HE and its association with serum zinc levels. *: Statistically significant. DCLD = decompensated chronic liver disease; HE = hepatic encephalopathy

Classification per West Haven criteria	Number of patients	Serum zinc level	P-value
		Mean ± SD	
Grade 1 HE	35	48.4 ± 7.7	0.000^*^
Grade 2 HE	37	40.7 ± 7.9	
Grade 3 HE	17	31.2 ± 4.8	
Grade 4 HE	11	29.0 ± 4.7	

On classifying the patients per the Child-Pugh scoring system, 99 (99.0%) patients were in Class C, implying decompensated disease and one (1.0%) was in Class B, implying functional compromise with none of the patients in Class A. On comparing the serum zinc levels across the Child-Pugh classes, the difference was found to be significant (p = 0.000), implying a significant decrease in serum zinc levels with increasing severity of the disease (Table 5).

**Table 5:** Classification of the DCLD patients per the Child-Pugh scoring and its association with zinc levels. *: Statistically significant. DCLD = decompensated chronic liver disease

Classification per Child-Pugh scoring	Number of patients	Serum zinc level, mean ± SD	P-value
Class B	1	53.6	0.000*
Class C	99	40.4 ± 9.9	

On classifying the patients per the MELD score, the majority (56%) had scores between 26 and 30, followed by scores more than 30 among 23% of patients (Table 6). On comparing the serum zinc level across the MELD score groups, the difference was found to be statistically significant, implying a significant decrease in serum zinc levels with increasing MELD score (Table 6).

**Table 6:** Classification of the DCLD patients per the MELD scoring and its association with serum zinc levels. *: Statistically significant. DCLD = decompensated chronic liver disease; MELD = Model for End-stage Liver Disease

MELD Score	No. of patients	Serum zinc level Mean ±SD	p-value
21 to 25	21	43.2±11.2	0.006*
26 to 30	56	41.7±9.2	
>30	23	35.0±9.3	

Pearson’s correlation coefficient (r) for assessing the correlation between serum zinc and serum albumin was 0.8939 with a corresponding p-value of <.00001, implying a strong positive correlation between the two variables.

## Discussion

Zinc, the second most abundant intracellular trace element, performs vital functions in the body, and its insufficiency deranges several organs and systems of the body. Although CLDs are regarded as diseases with a high risk of zinc depletion, this association has not been clearly explained [21].

In this study, we investigated 100 patients with decompensated cirrhosis of the liver/DCLD/liver cirrhosis and 50 healthy age and sex-matched controls. The most common etiology of DCLD was alcohol abuse (66%), followed by hepatitis B (23%) and others (10%). A study by Meena et al. [22] among 75 patients with DCLD also found alcohol abuse (90.7%) to be the most common etiology, followed by hepatitis B virus (10%), similar to our findings.

In our study, the serum zinc level of patients with DCLD was found to be significantly lower compared to that of healthy controls (p < 0.0001), implying a significant association of low serum zinc level with DCLD. Serum zinc level was also compared across the grades of hepatic encephalopathy, classified using West Haven Criteria [20]. Serum zinc level was significantly lower in patients with higher grades of hepatic encephalopathy, implying an association of low serum zinc level with increased severity of hepatic encephalopathy. Studies by Meena et al. [22] and Alikkanakath et al. [23] also found significantly lower serum zinc levels in patients with DCLD. Loomba et al. [16] studied 55 patients with hepatic failure and encephalopathy, of whom 30 had acute, five had sub-acute, and 20 had CLDs, and compared their zinc levels with 20 age and sex-matched controls. Patients with liver failure and encephalopathy had significantly lower zinc levels compared to the controls, implying an association of hepatic encephalopathy with lower zinc levels. Meena et al. [22] also found that patients with higher grades of hepatic encephalopathy had lower serum zinc levels.

In our study, the serum zinc level was also found to be significantly lower among patients in the higher class of Child-Pugh classification and with higher MELD scores, implying an association of low serum zinc level with increased disease severity and poor prognosis in patients with DCLD. A significant association of low serum zinc level with higher Child-Pugh score (p = 0.001) was also observed in the study by Meena et al. [22]. A study by Kamani et al. [24] from Pakistan studied patients with viral cirrhosis and compared the serum zinc level between the Child-Pugh classes B and C and healthy controls. Zinc deficiency was observed in 60% of the patients in Class C compared to only 26% in Class B. Comparison of the mean serum zinc level between the three groups using ANOVA showed a highly significant statistical difference, and a significant inverse correlation was also found between the Child-Pugh score and serum zinc level [24].

Few clinical trials have evaluated the effect of zinc supplementation on clinical outcomes in patients with CLDs including improvement in hepatic encephalopathy. Reding et al. [25] in a double-blind, randomized placebo-controlled trial reported that short-term (for seven days) oral zinc supplementation improved the hepatic encephalopathy state in 22 patients with cirrhosis. Similarly, Katayama et al. [26] in a preliminary randomized controlled trial showed that daily supplementation with zinc acetate for three months in patients with liver cirrhosis was an effective and safe therapy for managing hyperammonaemia. A systematic review and meta-analysis assessing the effect of oral zinc supplementation in patients with hepatic encephalopathy found a significant improvement in the performance of patients on the psychological test. However, zinc therapy was not found to be associated with a reduction in encephalopathy recurrence [27]. Similarly, a recent systematic review and meta-analysis assessing the effect of zinc treatment on clinical outcomes in patients with cirrhosis found that zinc supplementation is not associated with reduced mortality in patients with cirrhosis. However, findings may have been limited by the small number of included studies and significant heterogeneity among the studies [28].

Study limitations

This study had a few limitations. Being a single-center study is one of them. The small sample size, recruitment of controls from the same study site as cases, and differences in case mix are other limitations that may have implications on the study findings. There are circadian variations in the serum zinc levels, they are found to be high in the early morning and start decreasing toward the afternoon and evening [29,30]. Therefore, sample collection for estimating serum zinc levels should ideally be performed in the early morning hours when patients are in a fasting condition. However, because of the feasibility of collecting samples from all patients at the same time, this could not be ensured uniformly, and blood samples from a few patients were collected in the late morning or in the afternoon.

## Conclusions

Serum zinc levels in patients with decompensated cirrhosis of the liver are significantly reduced, and low serum zinc level is associated with increased severity of the disease. Serum zinc level was significantly lower among patients with higher grades of hepatic encephalopathy, implying that low zinc level may be a precipitating factor for hepatic encephalopathy. Furthermore, supplementation with zinc may possibly reduce the clinical worsening in such patients. Identification and treatment of underlying triggers and/or use of prophylactic lactulose/rifaximin in patients with low zinc levels in decompensated liver disease for preventing hepatic encephalopathy need to be studied further. Therefore, more research-based evidence is required through conducting well-designed randomized controlled trials to elaborate on the usefulness of correcting low serum zinc levels in patients with decompensated cirrhosis to prevent further worsening of the disease and the development of encephalopathy.
